# Complete Genome Sequence of a Highly Divergent Dengue Virus Type 2 Strain, Imported into Australia from Sabah, Malaysia

**DOI:** 10.1128/genomeA.00546-17

**Published:** 2017-07-20

**Authors:** Alyssa T. Pyke, Bixing Huang, David Warrilow, Peter R. Moore, Jamie McMahon, Bruce Harrower

**Affiliations:** Public Health Virology Laboratory, Forensic and Scientific Services, Coopers Plains, Queensland, Australia

## Abstract

In 2015, a female patient returning to Australia from Sabah, Malaysia, was diagnosed with a suspected sylvatic dengue virus type 2 (DENV-2) infection, becoming the second case of imported highly divergent dengue virus infection recorded in Australia. We describe here the complete genome sequencing of the DENV-2 strain isolated from this patient.

## GENOME ANNOUNCEMENT

Despite global efforts to mitigate infectious mosquito-borne viral diseases, millions of people are afflicted annually with dengue disease. The four dengue virus (DENV) serotypes (DENV-1 to DENV-4) are widely distributed throughout tropical and subtropical regions wherever *Aedes aegypti* and *Aedes albopictus* mosquito vectors are prevalent. Infection can range from a mild, febrile illness to more serious syndromes, including dengue hemorrhagic fever and dengue shock syndrome (1, 2). Belonging to the genus *Flavivirus* and family *Flaviviridae*, DENVs contain a 5′-capped, positive-sense, single-stranded RNA genome of approximately 11 kb encoding a single open reading frame (ORF) flanked by terminal 5′ and 3′ untranslated regions (UTRs). Translation and subsequent processing of the ORF yields three structural and seven nonstructural proteins (3).

The transmission of DENV involves both endemic/epidemic cycles between humans and *A. aegypti* or *A. albopictus* mosquitoes and sylvatic cycles between arboreal *Aedes* mosquitoes and nonhuman primates. While most human infections result from endemic/epidemic DENV transmission, spillover and infection of humans with sylvatic DENVs can occur. Current DENV-1 to DENV-4 serotypes are believed to have evolved independently from ancestral sylvatic progenitors given the basal positioning of sylvatic strains within phylogenetic trees (4–11).

Increased urbanization and deforestation has contributed to stochastic spillover and human infection with sylvatic DENV (4, 12). Transport via viremic travelers and importation into regions in which DENV is not endemic has also increased global dispersion of sylvatic strains and risk to public health (6, 8). In 2014, we isolated a highly divergent DENV-1 of likely sylvatic origin from a traveler returning from Brunei, Borneo, to Brisbane, Queensland, Australia (6). In 2015, we diagnosed the second Australian case of highly divergent DENV infection in a febrile female patient who had returned to Brisbane from Sabah, Malaysia, and were the first to report the discovery and isolation of the most divergent DENV-2 strain yet reported (D2Sab2015) (13).

We next determined the genome sequence of D2Sab2015 (passage 3 in C6/36 cells), including the 5′/3′ terminal sequences, using a rapid single RNA template methodology. Briefly, D2Sab2015 RNA was circularized using a strategy similar to that used previously (14). The 5′ m7GpppN cap was first removed using tobacco decapping enzyme (product 87 [Enzymax LLC, USA]). The decapped RNA was then ligated using T4 RNA ligase (New England Biolabs, USA). To obtain the complete genome sequence, the circularized RNA was then used in the Illumina sequencing platform as previously described (15). We also confirmed the Illumina derived 5′ and 3′ terminal UTR sequences using reverse transcription-PCR and Sanger dideoxy sequencing.

Although sequencing of the highly divergent DENV-2 has been described by others (GenBank accession no. KX274130) (16), our rapid approach combines Illumina sequencing with RNA circularization to obtain the whole genome, including the terminal sequences in one method. Further, we identified an additional 3′ terminal nucleotide base (T) in the D2Sab2015 sequence (total genome 10,737 nucleotides) compared to KX274130, which shares 100% nucleotide identity with D2Sab2015 for the remaining genomic sequence.

### Accession number(s).

The whole-genome sequence of DENV-2 strain D2Sab2015 has been deposited in GenBank under the accession number KY923048.
